# Supplementary material for: Deep phenotypic characterization of immunization-induced antibacterial IgG repertoires in mice using a single-antibody bioassay
Source: Commun Biol. 2020 Oct 26;3:614. doi: 10.1038/s42003-020-01296-3 (PMC7589517; doi:10.1038/s42003-020-01296-3)
Supplement: Supplementary file 1 — Supplementary Information [file 42003_2020_1296_MOESM1_ESM.pdf]

Supplementary information.

Deep phenotypic characterization of immunization-induced antibacterial IgG repertoires in mice using an antibody-resolution bioassay

Millie Heo[1], Guilhem Chenon[1], Carlos Castrillon[2,3,4], Jérôme Bibette[1], Pierre Bruhns[2], Andrew D. Griffiths[3], Jean Baudry[1], Klaus Eyer[1,5*]

[1] ʹLaboratoire Colloïdes et Matériaux Divisés’ (LCMD), ESPCI Paris, PSL Research University, CNRS UMR8231 Chimie Biologie Innovation, F-75005 Paris, France.

[2] Unit of Antibodies in Therapy and Pathology, Institut Pasteur, UMR1222 INSERM, F-75015 Paris, France.

[3] ‘Laboratoire de Biochimie’ (LBC), ESPCI Paris, PSL Research University, CNRS UMR8231 Chimie Biologie Innovation, F-75005 Paris, France.

[4] Sorbonne Université, Collège doctoral, F-75005 Paris, France

[5] Laboratory for Functional Immune Repertoire Analysis, Institute of Pharmaceutical Sciences, D-CHAB, ETH Zürich, Zürich.

[*] Corresponding author

Supplementary Table 1. List of the antibodies used in the study

Host	Host/Isotype	Reported antigen	Supplier and cat. no.
Mouse	IgG	LPS; E. coli J5	ThermoFisher Scientific MA 183152
Mouse	IgG	Not reported; P. aeruginosa	ThermoFisher Scientific MA1-83430
Mouse	IgG	LPS; S. typhimurium	Abcam ab8274
Mouse	IgM	Not reported; S. aureus	Merck Millipore/SigmaAldrich MAB930
Mouse	IgM	Not reported, used as isotype control	ThermoFisher Scientific MA110438
Rabbit	F(ab')$_2$ IgG-Fragment	Murine Fc IgG heavy chain specific	Jackson ImmunoResearch 315-606-046, Alexa647 labelled
Rabbit	IgG	µ chain IgM specific	Tebu Bio 221W99020C, Alexa555 labelled
Mouse	IgG	Ovalbumin	ThermoFisher HYB 099-11-02
Mouse	IgG	Ovalbumin	ThermoFisher HYB 099-09-02
Mouse	IgG	Ovalbumin	ThermoFisher HYB 099-02-02

Modelling of antibody binding to immobilized bacteria. To investigate the behaviour of the assay, we set up a theoretical model, similar to the model employed in reference[1]. Here, we directly immobilized the antigens on the beadline, thereby the fluorescence relocation was directly related to the affinity of the antibody; and only antibodies that recognize an epitope on the heat-killed bacteria (HK-B) would result in fluorescence relocation. The immobilized HK-B expressed a variety of different potential antigen molecules, mostly proteins, lipids and sugars, on their surface. Due to the unknown epitope of each antibody, the in-droplet concentration of the antigen could not be assumed as constant; but must be assumed to be highly variable. This variability will in turn influence the capacity of the nanoparticles to relocate antibody, and also resulted in different measured fluorescence relocation experimentally (see also Figure 2B, C for experiments). For simplicity, the model was based on the assumption that the binding of anti-IgG F(ab')2, antigen and antibody were iso-stoichiometric (1:1:1). Whilst the binding of a single anti-IgG $F(ab')_2$ to one IgG has been assayed[1], the binding of the antibody to the epitope could be either monovalent or bivalent, and therefore defined by affinity (1:1) or avidity (1:2). For modelling, this binding step would simply decrease K_{Dapp}, i.e. result in lower apparent dissociation constant due to bivalent binding. However, the parameters and equations were not influenced by this observation for general modelling.

For modelling, we first defined the present equilibria in the bactoline assay (SI Figure 2). In the equilibria in SI Figure 2,

S_0 represents the total concentration of immobilized bacterial antigen (HK-B),
x_0 the total concentration of IgG,
x the concentration of immobilized antibody bound to the antigen,
y_0 the total concentration of anti-IgG F(ab')2,
β the concentration of complex of F(ab')2 and antibody in solution, and
α the formed complex F(ab')2-IgG on the bacteria (i.e. that results in fluorescence relocation).

As in the experimental assays, fluorescence relocation was defined as α/y_0. This lead to the three different equations for the equilibria depicted in panels (a-c):

$$K_d = \frac{(x_0 - x)(S_0 - x)}{x} \quad (a)$$

$$K_{d1} = \frac{(x_0 - x - \beta)(y_0 - \alpha - \beta)}{\beta} \quad (b)$$

$$K_{d2} = \frac{(x - \alpha)(y_0 - \alpha - \beta)}{\alpha} \quad (c)$$

The unique plausible analytical solution for the equilibrium concentrations of x, (IgG bound to the magnetic nanoparticles), α (anti-IgG (ab')2 bound to IgG complexed with beads) and β (anti-IgG bound to free antibody) are given in equations 1-3:

$$x = \frac{1}{2}\left[(K_d + S_0 + x_0) - \sqrt{-4\,S_0\,x_0 + (K_d + S_0 + x_0)^2}\right] \quad (1)$$

$$\propto = \frac{x\ \left(K_{d1} + y_0 + x_0 - \sqrt{(K_{d1} + x_0)^2 + 2\,(K_{d1} - x_0)\,y_0 + y_0^2}\right)}{2\,x_0} \quad (2)$$

$$\beta = -\frac{(x - x_0)\,(K_{d1} + y_0 + x_0 - \sqrt{(K_{d1} + x_0)^2 + 2\,(K_{d1} - x_0)\,y_0 + y_0^2})}{2\,x_0} \quad (3)$$

Equations 1, 2 and 3 were used to model the bactoline bioassay. The default values of concentrations and dissociation constants used for simulations can be found in SI Table 2. In the modal, we wanted to study the influence of a varying antigen concentration and affinity on the position of the maximal fluorescence relocation; as it was used in experiments to estimate affinity and antigen availability. In the following, y_{max} is the maximal fluorescence relocation and x_{max} is the position of this maximum on the x-axis.

Supplementary Table 2: Default values used for the simulation of the assay.

Interaction	Kd [nM]	Concentration [nM]
F(ab')2 A647 anti-IgG Fc	15 nM	45 nM
Antigen	-	Variable, or 1 nM
Anti-antigen IgG	Variable, or 1 nM	From 0.1 to 250 nM

First modelling was performed for a constant affinity of 1 nM, but a variable in-droplet concentration of antigen from 1 pM to 52 nM (SI Figure 3A). The maximal fluorescence relocation value (y_{max}) and the concentration at which its achieved (x_{max}) were both found to be dependent on the antigen concentration used for modelling (Si Figure 3A and 4A). The maximal fluorescence relocation (y_{max}) was expected to increase with antigen concentration since antigen concentration directly defined the capacity of the bactoline (SI Figure 3A). With a higher capacity, higher amounts of antibody were able to relocate on the bactoline; and higher fluorescence relocation were expected. This was also observed in experiments with different amounts of antigen (ovalbumin) (Figure 2B). The non-linear relationship in SI Figure 3A was explained by the finite concentration of detection antibody (45 nM in-droplet concentration) that also limits the maximal fluorescence relocation in the bioassay (as it was also seen experimentally). Furthermore, the location of the maximum fluorescence relocation along the x-axis was modelled to be linearly dependent on the concentration of antigen as well; at least for the modelled concentration range.

Next, we varied affinity (5-100 nM) but left the antigen concentration constant (1 nM). Here, we found that y_{max} was not significantly altered by a variation of affinity (SI Figure 3B); a fact that was also experimentally confirmed (Figure 2C, D). Although a minor influence was measured and also modelled for, the influence of affinity on y_{max} was found to be negligible when compared to the influence of antigen concentration (100-1000 fold smaller); and was even smaller than the experimental noise of our measurements. On the other hand, we found that the location of said maxima on the x-axis (x_{max}) was strongly dependent on the used affinity for modelling.

From this model, we concluded that the maximal fluorescence relocation (y_{max}) was only significantly depending on the available antigen present within the droplet. However, x_{max} was found to be dependent on antigen concentration as well as affinity, and both values would be not known in a single-cell assay for each individual antibody. To circumvent this issue, we divided x_{max} by the number of available antigens, which is the y_{max}. By doing so, the influence of the antigen concentration was removed, and the resulting parameter, α (SI Figure 4C), solely correlated with affinity but no longer with antigen concentration (data not shown). Indeed, the shape of the curve closely matched the experimental and calibrated data (Figure 2E). Therefore, the location of said maximal fluorescence relocation along the x-axis (x_{max}), once corrected for antigen capacity, correlated with affinity only and could be used to extract an estimation of affinity. We confirmed the relationships found in the modelling experimentally (Figure 2 E and F).

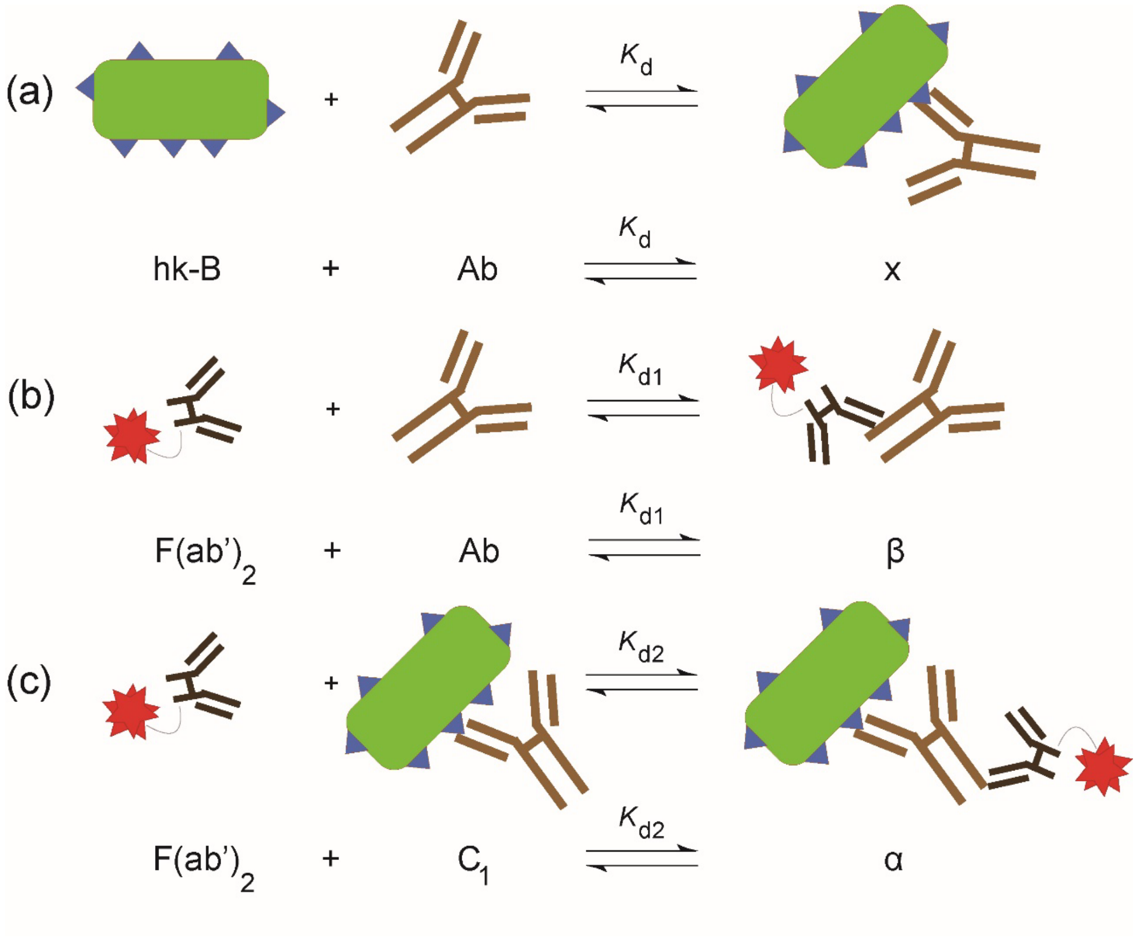

Supplementary Fig. 1. Equilibria in the bactoline assay. (a) Equilibrium between the bacterial surface antigen and the IgG. (b) Equilibrium between the anti-IgG F(ab')2 and the IgG in solution. (c) Equilibrium between the F(ab')2 and the immobilized IgG on the bacterial surface.

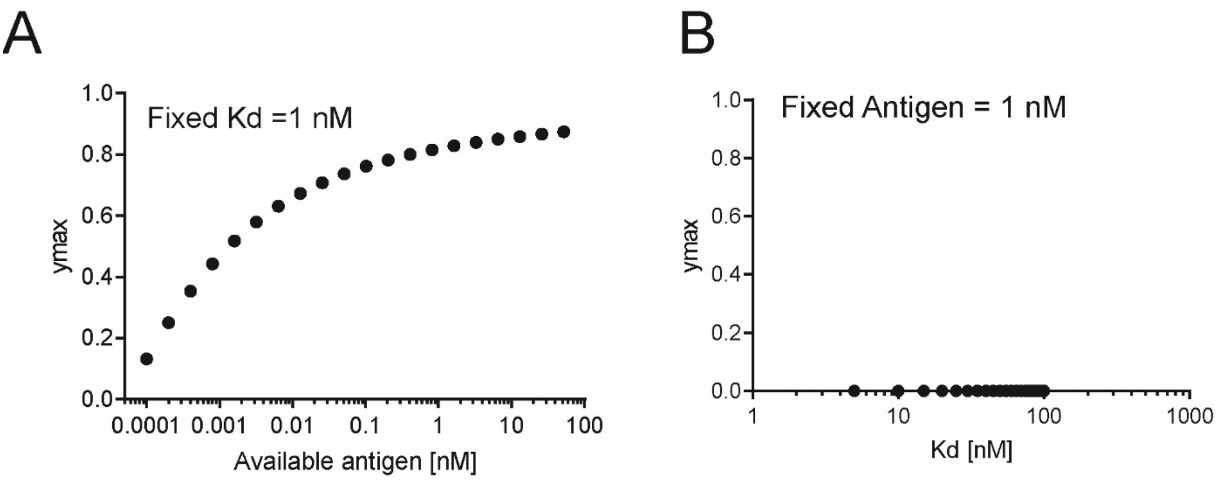

Supplementary Fig. 2. Modeling of the assay; y_{max}. A) Maximal fluorescence relocation (y_{max}) as a function of various antigen concentrations present within the droplet; and a constant affinity of 1 nM of the antibody. B) Maximal fluorescence relocation (y_{max}) as a function of various binding strengths; and a constant concentration of antigen 1nM within the droplet.

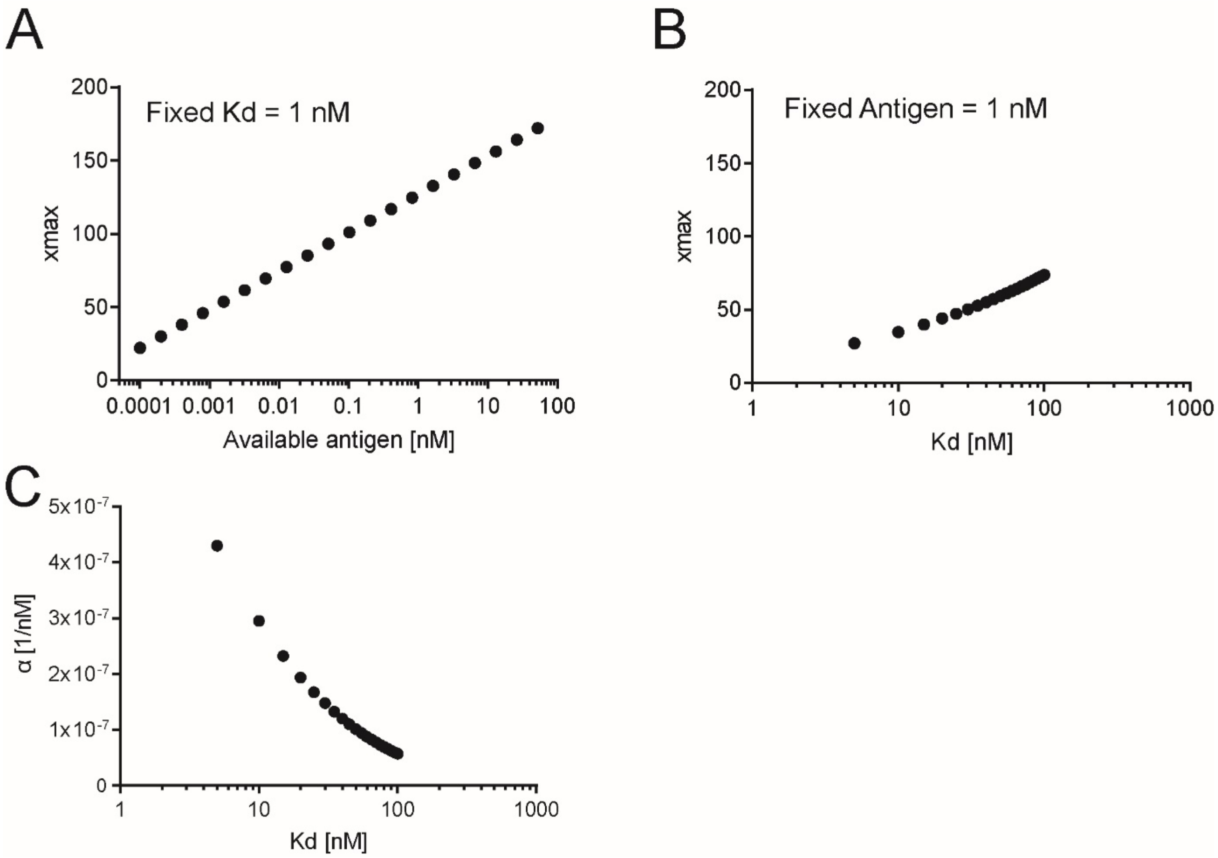

Supplementary Fig. 3. Modeling of the assay; x_{max} and α. A) The position of the maximal fluorescence relocation along the x-axis (x_{max}) as a function of various antigen concentrations present within the droplet. B) The position of the maximal fluorescence relocation along the x-axis (x_{max}) as a function of various apparent dissociation constants; and a constant concentration of antigen within the droplet. C) Factor α, i.e. x_{max} that has been corrected by y_{max}, correlated with binding strength (here dissociation constant). A similar relationship was found in calibration experiments (Figure 2E).

Multiplexing of IgM and IgG on the V_HH beadline. In addition to the bactoline, we utilized the beadline that are coated by anti-mouse kappa light chain VHH fragments[1], which bind to all mouse isotype antibodies. Of those antibodies, we were able to detect IgM and IgG antibodies by the two reporter antibodies: the anti-IgM mu chain specific rabbit antibodies conjugated to Alexa555 and the anti-IgG Fc chain specific rabbit antibodies conjugated to Alexa647.

Calibration curves were measured using commercial IgG and IgM antibodies; and showed that the used detection antibodies were specific for their respective isotypes (SI Figure 6). In the presence of murine IgM isotypes (SI Figure 6, left), increased Alexa555 fluorescent signal on the beadline was observed that was the result of relocation of the anti-IgM, whereas no fluorescence relocation was observed in the anti-IgG-Alexa647. Alternatively, concentration dependent fluorescence relocation in the Alexa647 channel (anti-IgG) was observed when IgG was added, while anti-IgM-Alexa 555 fluorescence beadline signal remained non-significantly different from the background signal (SI Figure 6, right). The relocated fluorescence signals on the beadline were dependent on the concentration of both IgG and IgM antibodies, allowing sensitive and quantitative measurements. In this study, 45nM of each anti-IgG and anti-IgM reporter antibodies were presented in droplet.

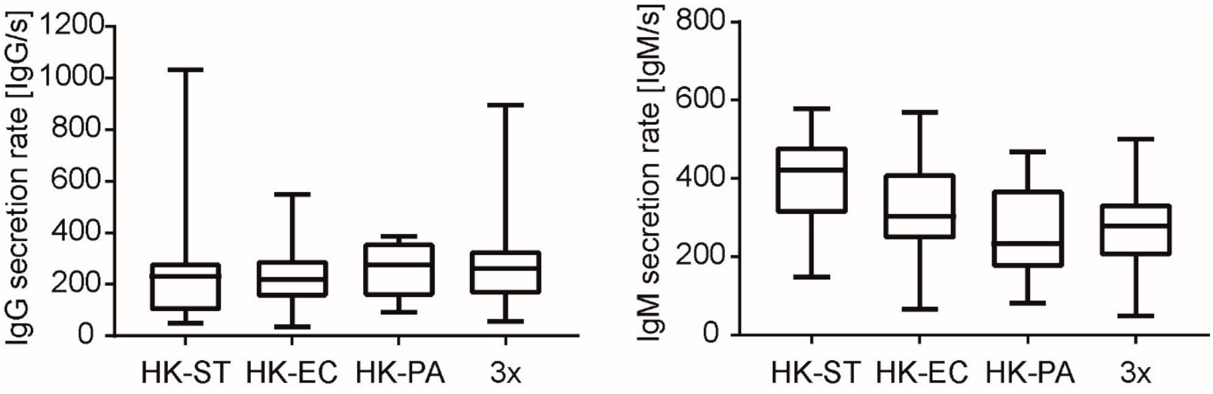

Supplementary Figure 4. A) Measured distribution of the IgG-secretion rates and B) of IgM-secreting rates. Median secretion rates and distributions were found to be non-significantly different for IgG; and only slightly different for IgM. All data points were performed in triplicates, measured in the spleen 6 days after secondary immunization.

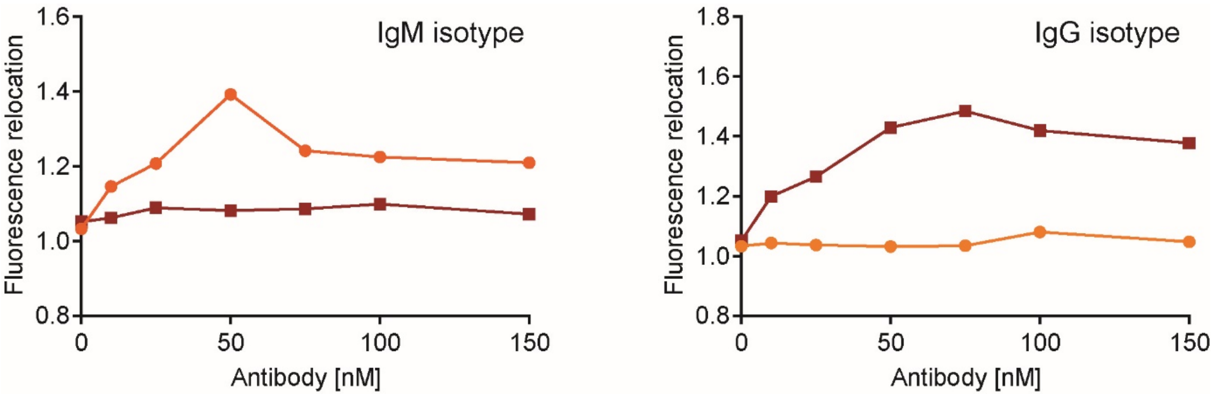

Supplementary Figure 5. Beadline immunoassay calibration for IgM and IgG. Right: Relocation of anti-IgM-Alexa555 (Orange) to the beadline as a function of the concentration of added IgM calibration antibody. Left: Relocation of anti-IgG-Alexa647 (Red) to the beadline as a function of the concentration of IgG antibodies in the presence of both 45 nM anti-IgG(Fc) as well as 45 nM anti-IgM reporter antibodies.

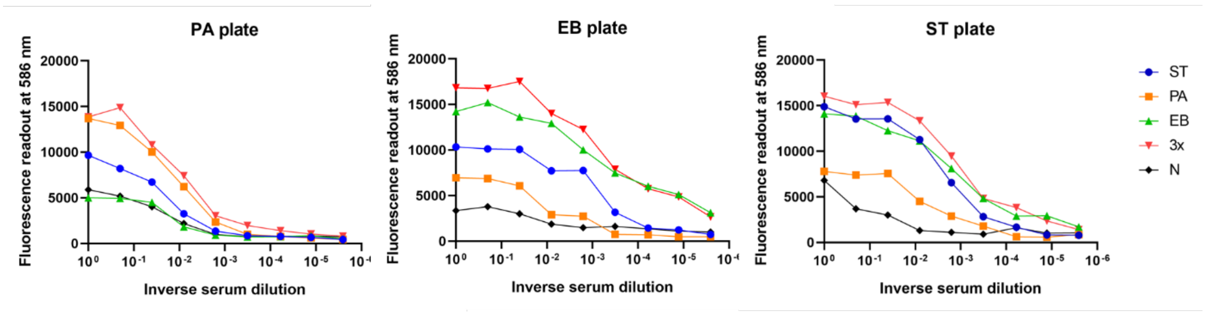

Supplementary Figure 6. Serum titer measurements of all five types of immunized mice using ELISA. Serum from all five differently immunized group of mice were tested against all three bacterial surfaces (PA plate, EB plate, ST plates).

References.

1. Eyer, K., et al., *Single-cell deep phenotyping of IgG-secreting cells for high-resolution immune monitoring.* Nat Biotechnol, 2017. **35**(10): p. 977-982.
