# Supplementary material for: Deep phenotypic characterization of immunization-induced antibacterial IgG repertoires in mice using a single-antibody bioassay
Source: Commun Biol. 2020 Oct 26;3:614. doi: 10.1038/s42003-020-01296-3 (PMC7589517; doi:10.1038/s42003-020-01296-3)
Supplement: Supplementary file 2 — Reporting Summary [file 42003_2020_1296_MOESM2_ESM.pdf]

nature research

Corresponding author(s): Eyer Klaus

Last updated by author(s): 12.08.2020

Reporting Summary

Nature Research wishes to improve the reproducibility of the work that we publish. This form provides structure for consistency and transparency in reporting. For further information on Nature Research policies, seeAuthors & Referees and theEditorial Policy Checklist .

Statistics

For all statistical analyses, confirm that the following items are present in the figure legend, table legend, main text, or Methods section.

n/a	Confirmed	
☐	☒	The exact sample size (*n*) for each experimental group/condition, given as a discrete number and unit of measurement
☐	☒	A statement on whether measurements were taken from distinct samples or whether the same sample was measured repeatedly
☐	☒	The statistical test(s) used AND whether they are one- or two-sided<br>*Only common tests should be described solely by name; describe more complex techniques in the Methods section.*
☐	☒	A description of all covariates tested
☒	☐	A description of any assumptions or corrections, such as tests of normality and adjustment for multiple comparisons
☐	☒	A full description of the statistical parameters including central tendency (e.g. means) or other basic estimates (e.g. regression coefficient) AND variation (e.g. standard deviation) or associated estimates of uncertainty (e.g. confidence intervals)
☐	☒	For null hypothesis testing, the test statistic (e.g. *F*, *t*, *r*) with confidence intervals, effect sizes, degrees of freedom and *P* value noted<br>*Give P values as exact values whenever suitable.*
☒	☐	For Bayesian analysis, information on the choice of priors and Markov chain Monte Carlo settings
☒	☐	For hierarchical and complex designs, identification of the appropriate level for tests and full reporting of outcomes
☒	☐	Estimates of effect sizes (e.g. Cohen's *d*, Pearson's *r*), indicating how they were calculated

Our web collection on statistics for biologists contains articles on many of the points above.

Software and code

Policy information about availability of computer code

Data collection	Commercial software from Nikon was used to aquire data [NIS-Elements (Version 4.50)].
Data analysis	Matlab (R2019a version 9.6, Image processing toolbox and VLFeat open source library), Custom DropMap Matlab scripts is available from GitHub repository, https://github.com/LCMD-ESPCI/dropmap-analyzer

For manuscripts utilizing custom algorithms or software that are central to the research but not yet described in published literature, software must be made available to editors/reviewers. We strongly encourage code deposition in a community repository (e.g. GitHub). See the Nature Research guidelines for submitting code & software for further information.

Data

Policy information about availability of data

All manuscripts must include a data availability statement. This statement should provide the following information, where applicable:

- Accession codes, unique identifiers, or web links for publicly available datasets
- A list of figures that have associated raw data
- A description of any restrictions on data availability

The original data used in this publication are made available in a curated data archive at ETH Zürich (https://www.research-collection.ethz.ch) under the DOI 10.3929/ethz-b-000431634.

Field-specific reporting

Please select the one below that is the best fit for your research. If you are not sure, read the appropriate sections before making your selection.

Life sciences study design

All studies must disclose on these points even when the disclosure is negative.

Sample size	Different experiments were analyzed to illustrate the potential of the single-cell analysis technology. For each experiment, 3 different mice were independently tested. The number of single-cells analyzed in each sample ranged from XX
Data exclusions	Data from droplets containing >1 cell were excluded from the analyses (as the aim of the study was to measure phenotypes of single-cells)
Replication	During the method development steps, duplicate experiments (same sample studied) were performed to ensure that results were reproducible at cell population level.
Randomization	No method of randomization was chosen.
Blinding	No blinding was performed.

Reporting for specific materials, systems and methods

We require information from authors about some types of materials, experimental systems and methods used in many studies. Here, indicate whether each material, system or method listed is relevant to your study. If you are not sure if a list item applies to your research, read the appropriate section before selecting a response.

Materials & experimental systems

n/a	Involved in the study	
☐	☒	Antibodies
☒	☐	Eukaryotic cell lines
☒	☐	Palaeontology
☐	☒	Animals and other organisms
☒	☐	Human research participants
☒	☐	Clinical data

Methods

n/a	Involved in the study	
☒	☐	ChIP-seq
☒	☐	Flow cytometry
☒	☐	MRI-based neuroimaging

Antibodies

Antibodies used	Mouse IgG anti- E. coli J5; ThermoFisher Scientific MA 183152<br>Mouse IgG anti- P. aeruginosa; ThermoFisher Scientific MA1-83430<br>Mouse IgG anti- S. typhimurium; Abcam ab8274<br>Mouse IgM anti- S. aureus; Merck Millipore/SigmaAldrich MAB930<br>Mouse IgM isotype control; ThermoFisher Scientific MA110438<br>Rabbit F(ab')$_2$ IgGFragment Murine Fc IgG heavy chain specific; Jackson ImmunoResearch 315-606-046, Alexa647 labelled<br>Rabbit IgG µ chain IgM specific; Tebu Bio 221W99020C, Alexa555 labelled<br>Mouse IgG anti-Ovalbumin; ThermoFisher HYB 099-11-02<br>Mouse IgG anti-Ovalbumin; ThermoFisher HYB 099-09-02<br>Mouse IgG anti-Ovalbumin; ThermoFisher HYB 099-02-02
Validation	Species and specificity of the antibodies was confirmed by ELISA measurements. The data is provided within the research paper and the supporting information.

Animals and other organisms

Policy information about studies involving animals; ARRIVE guidelines recommended for reporting animal research

Laboratory animals	Mus musculus, BALB/c, females, age 8 weeks at the start of the immunization, supplied by Janvier Laboratories
Wild animals	Not applicable.
Field-collected samples	Not applicable.
Ethics oversight	Experiments using mice were validated by the CETEA ethics committee number 89 (Institute Pasteur, Paris, France) under #2013-0103, and by the French Ministry of Research under agreement #00513.02.

Note that full information on the approval of the study protocol must also be provided in the manuscript.
